# Positive and Negative Symptoms in Schizophrenia Relate to Distinct Oscillatory Signatures of Sensory Gating

**DOI:** 10.3389/fnhum.2016.00104

**Published:** 2016-03-14

**Authors:** Julian Keil, Yadira Roa Romero, Johanna Balz, Melissa Henjes, Daniel Senkowski

**Affiliations:** Multisensory Integration Group, Department of Psychiatry and Psychotherapy, St. Hedwig Hospital, Charité – Universitätsmedizin BerlinBerlin, Germany

**Keywords:** inter-trial coherence, power, gamma-band, alpha-band, phenotype, cluster analysis

## Abstract

Oscillatory activity in neural populations and temporal synchronization within these populations are important mechanisms contributing to perception and cognition. In schizophrenia, perception and cognition are impaired. Aberrant gating of irrelevant sensory information, which has been related to altered oscillatory neural activity, presumably contributes to these impairments. However, the link between schizophrenia symptoms and sensory gating deficits, as reflected in oscillatory activity, is not clear. In this electroencephalography study, we used a paired-stimulus paradigm to investigate frequency-resolved oscillatory activity in 22 schizophrenia patients and 22 healthy controls. We found sensory gating deficits in patients compared to controls, as reflected in reduced gamma-band power and alpha-band phase synchrony difference between the first and the second auditory stimulus. We correlated these markers of neural activity with a five-factor model of the Positive and Negative Syndrome Scale. Gamma-band power sensory gating was positively correlated with positive symptoms. Moreover, alpha-band phase synchrony sensory gating was negatively correlated with negative symptoms. A cluster analysis revealed three schizophrenia phenotypes, characterized by (i) aberrant gamma-band power and high positive symptoms, (ii) aberrant alpha-band phase synchrony, low positive, and low negative symptom scores or (iii) by intact sensory gating and high negative symptoms. Our study demonstrates that aberrant neural synchronization, as reflected in gamma-band power and alpha-band phase synchrony, relates to the schizophrenia psychopathology. Different schizophrenia phenotypes express distinct levels of positive and negative symptoms as well as varying degrees of aberrant oscillatory neural activity. Identifying the individual phenotype might improve therapeutic interventions in schizophrenia.

## Introduction

A consistent observation in schizophrenia is that affected individuals are impaired in their ability to gate irrelevant sensory input (Patterson et al., 2008). It has been proposed that sensory gating deficits lead to a sensory overload, which contributes to cognitive deficits in schizophrenia (McGhie and Chapman, 1961; Shakow, 1963; Freedman et al., 1991; Bob et al., 2014). A commonly used experimental paradigm to study sensory gating is the auditory paired-stimulus paradigm, in which two identical stimuli are presented in close succession (Adler et al., 1982; Bramon et al., 2004). Electroencephalography (EEG) and magnetoencephalography (MEG) studies revealed that in healthy individuals the early cortical response to the second stimulus is considerably smaller than the response to the first stimulus (Clementz et al., 1997b; Hall et al., 2011; Popov et al., 2011). In schizophrenia patients, the response difference between the first and second stimulus is typically reduced (Patterson et al., 2008).

Surprisingly, only few studies have linked the sensory gating deficit in electrophysiological data to positive or negative symptoms in schizophrenia (Potter et al., 2005; Keshavan et al., 2008). Auditory gating deficits, as reflected in the P50 event-related potential (ERP), were found to be correlated with negative symptoms (Ringel et al., 2004; Louchart-de la Chapelle et al., 2005; Thoma et al., 2005). Moreover, the P50 auditory gating deficit has been correlated with the severity of auditory hallucinations (Smith et al., 2013). Measuring schizotypy in healthy individuals, a recent study revealed that the P50 auditory gating deficit relates to cognitive disorganization and impulsive non-conformity (Park et al., 2015). Taken together, these studies suggest a link between sensory gating deficits and the schizophrenia psychopathology.

In recent years, the focus of electrophysiological research has changed from the analysis of ERPs to a more complex view encompassing dynamics in cortical networks (Lisman, 2012; Uhlhaas and Singer, 2015). Numerous studies provided evidence that coordinated oscillatory activity in neural populations plays a role for cognitive processes (Singer, 1999; Senkowski et al., 2008; Uhlhaas and Singer, 2010). Moreover, aberrant oscillatory activity presumably contributes to the psychopathology of schizophrenia (Andreasen et al., 1999; Andreasen, 2000; Senkowski and Gallinat, 2015). Various studies reported deficits in oscillatory activity in this patient group (Leicht et al., 2010; Mathiak et al., 2011; Popov et al., 2011; see Lee et al., 2003a and Senkowski and Gallinat, 2015 for reviews). Interestingly, it has been hypothesized that different aspects of oscillatory activity are relevant for different symptoms in schizophrenia (Pittman-Polletta et al., 2015; Uhlhaas and Singer, 2015). Aberrant temporal synchronization might primarily contribute to cognitive impairments and negative symptoms (Andreasen et al., 1999; Andreasen, 2000). Although the evidence is less robust, negative symptoms have also been associated with reduced resting-state alpha-band power and increased low-frequency power (Sponheim et al., 2000; see Boutros et al., 2014 for a review). Furthermore, deficits in higher frequency oscillations might predominantly contribute to positive symptoms (Senkowski and Gallinat, 2015; Uhlhaas and Singer, 2015). However, results on resting-state as well as stimulus-evoked gamma-band activity are mixed (e.g., Gallinat et al., 2004; Brenner et al., 2009; Hong et al., 2012). Taken together, electrophysiological markers promise to provide important information for the identification of specific phenotypes of schizophrenia (Smucny et al., 2013; Ethridge et al., 2015).

Analyzing frequency-resolved oscillatory activity allows disentangling the influence of local synchrony, i.e., oscillatory power, from temporal synchronization, i.e., phase consistency over time. Hence, the analysis of oscillatory activity can provides important information on aberrant stimulus processing in schizophrenia (Brockhaus-Dumke et al., 2008). A number of studies suggested that stimulus-processing deficits in schizophrenia are reflected in amplitude and phase of various frequency bands (Jansen et al., 2004; Moran et al., 2012; Carolus et al., 2014). For instance, some studies reported a reduction in low-frequency power in the theta-band and alpha-band in sensory gating paradigms (Moran et al., 2012; Carolus et al., 2014). In addition to alterations in power, phase coherence in the same frequency ranges appears to be impaired in schizophrenia (Jansen et al., 2004; Brockhaus-Dumke et al., 2008). Thus far, however, only few studies have related time–frequency resolved cortical activity to the schizophrenia symptomatology. One study revealed a reduction in evoked gamma-band activity that was associated with altered auditory and visual perception (Johannesen et al., 2008). Another study found that reduced stimulus evoked beta-band power in sensory gating was related to negative symptoms in schizophrenia (Smucny et al., 2013). Furthermore, another study showed that reduced alpha-band power in sensory gating was associated with reduced overall level of functioning and reduced processing speed (Hong et al., 2012). To summarize, recent findings suggest that reduced power and phase coherence reflect sensory gating deficits in schizophrenia.

In the present study, we investigated the relationships between positive and negative symptoms in schizophrenia and sensory gating deficits as reflected in oscillatory activity. Previously, auditory sensory processing has been linked to gamma-band activity (Pantev et al., 1991; Tiitinen et al., 1993). Moreover, there is evidence that gamma-band activity plays a role in auditory hallucinations (Reulbach et al., 2007; Spencer et al., 2009). Therefore, we hypothesized that gamma-band power in auditory gating relates to positive symptoms in schizophrenia. Aberrant temporal synchronization has been shown to affect information processing (Andreasen et al., 1999; Koh et al., 2011). Therefore, we hypothesized a relationship between phase coherence in auditory gating and negative symptoms in schizophrenia. Finally, we predicted that individual symptom patterns would enable us to identify patient clusters that share common phenotypes. Exploring possible subgroups within schizophrenia could further our understanding of the underlying psychopathology (Boutros et al., 2014).

## Materials and Methods

### Participants

Twenty-two schizophrenia patients (ScZ, seven female, 37.23 ± 7.75 years) with the DSM-4 diagnosis schizophrenia were recruited from outpatient units of the Charité – Universitäts medizin Berlin. The psychiatric diagnosis was assessed by a senior psychiatrist at the recruiting institution. Twenty-two education, handedness, gender, and age matched healthy control participants (HC, eight female, 37.09 ± 8.47 years), who were screened for psychopathology with the German version of the Structured Clinical Interview for DSM-4-R Non-Patient Edition (SCID), were recruited from the general population (**Table 1**). In all participants the Brief Assessment of Cognition in Schizophrenia (BACS) was assessed (Keefe, 2004). Severity of symptoms was obtained by the Positive and Negative Syndrome Scale by trained clinicians (PANSS; Kay et al., 1987). In accordance with a 5-factor model, items were grouped into factors “positive,” “negative,” “depression,” “excitement,” and “disorganization” (Wallwork et al., 2012). All participants gave written informed consent, had normal hearing and normal or corrected to normal vision, and no record of neurological disorders. No participant met DSM-4-R criteria for alcohol or substance abuse. A random sample of 45% of participants underwent a multi drug-screening test. The study was performed in accordance with the Declaration of Helsinki and the local ethics commission approved the study.

**Table 1:** Overview of demographic data.

	ScZ		HC		Statistics	
	Mean	*SD*	Mean	*SD*	*t*-values	*p*-values
Age (years)	37.23	7.75	37.09	8.47	0.06	0.96
Education (years)	11.00	1.72	11.09	1.6	-0.18	0.86
Daily cigarettes	4.27	3.41	2.72	3.58	1.46	0.15
Illness duration (years)	9.18	5.03	–	–	–	–
Chlorpromazine eq.	387.70	200.45	–	–	–	–
	
	***N***		***N***			
	
Gender (m/f)	15/7		14/8			
Handedness (r/l)	19/3		20/2			
Antipsychotic med.	22		–			
Haloperidol	1		–			
Amisulpride	8		–			
Clozapine	4		–			
Quetiapine	2		–			
Olanzapine	6		–			
Aripiprazole	3		–			
Risperidone	4		–			
Paliperidone	1		–			
Antidepressive med.	4		–			
Mirtazapine	1		–			
Escitalopram	2		–			
Paroxetine	1		–			

	**Mean**	***SD***	**Mean**	***SD***	***t*-values**	***p*-values**

**BACS**						
Verbal memory	42.90	12.63	48.32	10.80	-1.527	0.134
Digit	19.50	4.23	20.90	3.74	-0.171	0.248
Motor	67.18	11.42	76.59	10.68	-2.822	0.007
Fluency	48.59	13.86	54.18	15.93	-1.242	0.221
Symbol coding	55.14	13.28	57.09	13.44	-0.485	0.630
ToL	18.14	2.64	17.95	2.30	0.244	0.809
Total score	251.45	40.13	275.05	37.17	-2.023	0.050
**PANSS**						
Positive factor	10.10	1.92	–	–	–	–
Negative factor	15.20	2.50	–	–	–	–
Disorganized factor	7.43	1.78	–	–	–	–
Excited factor	8.05	1.12	–	–	–	–
Depressed factor	8.14	1.31	–	–	–	–

*Note that most patients received more than one antipsychotic medication, and four patients received additional antidepressive medication. No mood stabilizers, anticonvulsants or sedatives were prescribed.*

### Experimental Design

One hundred S1–S2 stimulus pairs of 0.003 s white noise were presented with a 0.5 s onset-to-onset interstimulus interval. Stimulus pairs were presented with a random intertrial interval of 7–9 s (mean = 8 s) at 65 dB (SPL) via a single mono speaker (Bose Companion 2) in a sound-attenuating, electrically shielded chamber. Participants were instructed to attend to auditory stimuli and were asked to keep their gaze on a small fixation cross, which was shown on a display placed in front of them.

### EEG Recording and Data Analysis

Data were recorded using a 128 channel active EEG system (EasyCap, Herrsching, Germany), which included two EOG electrodes (online: 1000 Hz sampling rate with a 0.016–250 Hz bandpass filter; oﬄine: downsampling to 500 Hz, 1–125 Hz FIR bandpass filtering and 49.1–50.2 Hz, fourth order Butterworth notch filtering). To correct for EOG and ECG artifacts, independent component (IC) analyses were conducted (Lee et al., 1999). On average 12.12 ± 3.80 ICs for ScZ and 13.95 ± 4.73 ICs for HC were rejected based on visual inspection (Chaumon et al., 2015). Remaining noisy channels were interpolated using spherical interpolation (ScZ = 15.14 ± 4.29 channels; HC = 13.55 ± 5.08 channels). Analysis of EEG data focused on the comparison of oscillatory responses to paired auditory stimuli. Additionally, we analyzed the auditory evoked P50 component (see Supplementary Material). Data were re-referenced to common average and split into trials of –1 s to 3 s around the S1 onset and those containing muscular artifacts or amplitudes of ±100 μV were rejected by visual inspection. On average, 59.32 trials for ScZ and 60.68 trials for HC were used for the further analyses. To estimate oscillatory power and temporal synchronization, single-trial data were transformed into the time–frequency domain using a multitaper approach (Mitra and Pesaran, 1999). Spectral estimates were calculated on sliding time–frequency windows (8–80 Hz, window size = 3 cycles per frequency, ±3.5 Hz frequency smoothing) based on a discrete prolate spheroid sequence of tapers with a step size of 0.025 s and 2 Hz. Single-trial power was normalized to reflect the relative change from baseline (–0.5 s to –0.1 s baseline window). As a measure of temporal synchronization, phase coherence across trials, i.e., inter-trial coherence (ITC), was computed on single-trial Fourier values from the same time–frequency transformation without baseline correction (Cheron et al., 2007). EEG data analysis was performed using EEGLab (Delorme and Makeig, 2004) and FieldTrip (Oostenveld et al., 2011).

### Statistical Analysis

Transient auditory stimulation typically induces a mediocentral increase in oscillatory activity between 0.05 and 0.1 s after stimulus onset (Clementz et al., 1997b; Leicht et al., 2010; Popov et al., 2011). Therefore, we extracted 0.05 s intervals starting 0.05 s after the onset of the S1 and S2 from the power and ITC time–frequency data. In a first step, we computed sensory gating within each participant. As a measure of normalized sensory gating for single-trial power, we computed dependent-samples *t*-tests for each time–frequency tile for S2 versus S1 within each participant. ITC is computed across trials; therefore we used the S2–S1 difference as a measure of sensory gating. In a second step, power and ITC sensory gating values in whole frequency range of 8–80 Hz without *á priori* restriction to frequency bands were compared between groups. To this end, independent-samples *t*-tests with Monte–Carlo randomization and cluster-based correction for multiple comparisons were used (Maris and Oostenveld, 2007). This approach allows the identification of clusters of significant effects in three-dimensional space (i.e., time, frequency, and sensor), effectively controlling for multiple comparisons. To elucidate, whether ScZ and HC show different oscillatory activity following S1 and S2, additional repeated measures ANOVAs with the factors Group (ScZ vs. HC) and Stimulus (S1 vs. S2), as well as appropriate *post hoc t*-tests were computed (see Supplementary Material). Pearson correlations were computed between psychopathology scores (i.e., PANSS), oscillatory power, and ITC. S2–S1 sensory gating measures in power and ITC were tested for normality using Shapiro–Wilk tests. In the next step, oscillatory power and ITC were averaged over the clusters obtained in the group analysis. One patient was identified as an outlier (>2.5 SD) based on the PANSS positive factor and was therefore excluded from further analysis. Since PANSS symptom scores were grouped into five factors, a Bonferroni-corrected alpha-level of 0.05/5 = 0.01 was used for the two-tailed correlation between psychopathology scores and EEG data. To statistically control for the influence of antipsychotic medication, medication dosage was converted to chlorpromazine equivalent level (Gardner et al., 2010), and entered as covariate to partial correlation analyses in ScZ. Moreover, to explore whether there are possible schizophrenia phenotypes, a k-means clustering analysis was used. This was done to identify patient clusters with common symptoms in 4-dimensional space based on power, ITC, PANSS positive, and negative factor (Schear, 1987; Olbrich et al., 2012). To this end, time–frequency power and ITC were averaged over the clusters obtained in the group comparison and data were sorted into an increasing number of clusters (i.e., the number of subjects for which all data were available minus one). Afterward, the optimal number of clusters was identified using the Akaike Information Criterion (AIC). Correlation and k-means cluster analyses were performed using R (R Development Core Team, 2011).

## Results

### Stimulus-Induced Oscillatory Power

**FIGURE 1:**
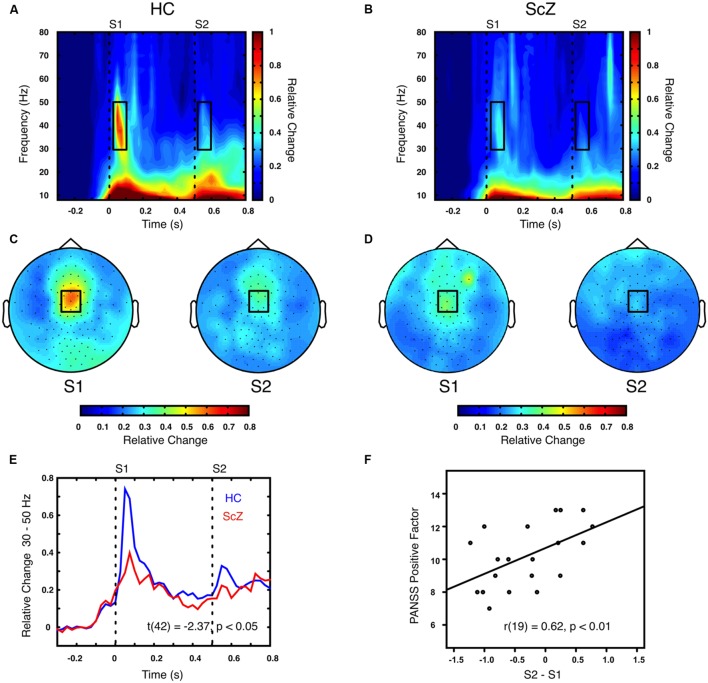
**Time–frequency representation of oscillatory power induced by the paired S1–S2 stimuli for HC and ScZ. (A,B)** Depict changes in oscillatory power relative to baseline in mediocentral electrodes for HC and ScZ, respectively. Dashed lines indicate the onset of the auditory stimuli. Black boxes mark the time–frequency cluster (0.05–0.1 s; 30–50 Hz) in which a significant auditory gating difference was found between groups. **(C,D)** Depict the topography of S1 and S2 for the time–frequency window marked in **(A,B)**. Black boxes mark the mediocentral EEG electrode cluster, in which a significant auditory gating difference was found between groups depicted in **(A,B)**. **(E)** Illustrates the time-course of mediocentral 30–50 Hz frequency window for HC (blue) and ScZ (red). **(F)** Illustrates the significant positive correlation between auditory gating in gamma-band power and the PANSS positive factor for ScZ.

Auditory gating was defined as the difference in power increase following two paired auditory stimuli (i.e., S2–S1). The S2–S1 difference between ScZ and HC was statistically assessed using independent-samples *t*-tests. In both groups, the S2 (mean ± SD, ScZ: 0.21 ± 0.11, HC: 0.30 ± 0.22) induced weaker power responses than the S1 (ScZ: 0.34 ± 0.26, HC: 0.63 ± 0.51), leading to negative S2–S1 gating differences. The gamma-band power gating difference at a mediocentral electrode cluster was significantly stronger in HC than in ScZ [30–50 Hz, *t*(42) = 2.37, *p* < 0.05, **Figures 1A–E**]. Additional ANOVAs and *t*-tests indicated that gamma-band power was not significantly different between ScZ and HC following S1 or S2 (see Supplementary Material). The S2–S1 gamma-band power difference was distributed normally (*W* = 0.96, *p* = 0.55). Within ScZ the gamma-band power gating difference correlated significantly with the PANSS positive factor [*r*(19) = 0.62, *p* < 0.01, **Figure 1F**]. The correlation remained significant when controlling for medication [*r*(18) = 0.57, *p* < 0.01]. **Tables 2** and **3** summarize the main findings.

**Table 2:** Overview of power and ITC effects.

Metric	Stimulus	ScZ Mean	ScZ SD	HC Mean	HC SD	*t*-value	*p*-value
Gamma-Band	S1	0.34	0.26	0.63	0.51	2.38	<0.05
Power	S2	0.21	0.11	0.30	0.22	1.78	0.08
(30–50 Hz)	S2–S1	-0.37	0.72	-0.95	0.87	2.37	<0.05
Alpha-Band	S1	0.22	0.05	0.33	0.10	4.25	<0.001
ITC	S2	0.17	0.04	0.19	0.06	1.08	0.29
(8–12 Hz)	S2–S1	-0.08	0.09	-0.19	0.13	3.24	<0.01

*Mean and SD values were computed over the sensor-time–frequency clusters obtained from the group comparison.*

**Table 3:** Overview of correlations between power, ITC, and PANSS factors.

Metric	PANSS-factors	*r*(19)	*p*
Gamma-Band Power	Positive	0.62	<0.005
(30–50 Hz)	Negative	-0.27	0.24
	Disorganized	-0.23	0.31
	Excited	0.14	0.55
	Depressed	0.16	0.48
Alpha-Band ITC	Positive	0.25	0.27
(8–12 Hz)	Negative	-0.54	<0.01
	Disorganized	-0.37	0.09
	Excited	0.04	0.83
	Depressed	0.26	0.25

*Power and ITC values entered into the correlations were computed over the sensor-time–frequency clusters obtained from the group comparison.*

### Inter-Trial Coherence

**FIGURE 2:**
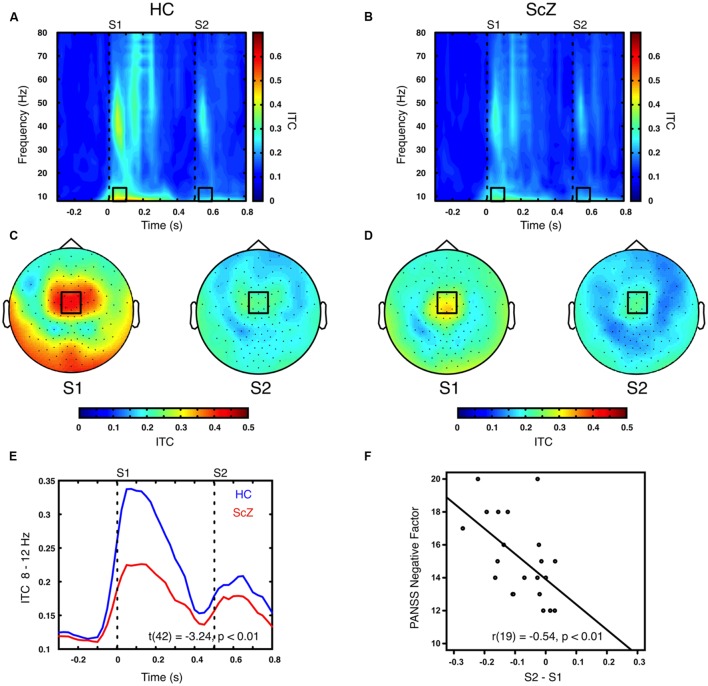
**Time–frequency representation of inter-trial coherence (ITC) induced by the paired S1–S2 stimuli for HC and ScZ. (A,B)** Depict changes in ITC in mediocentral electrodes for HC and ScZ, respectively. Dashed lines indicate the onset of the auditory stimuli. Black boxes mark the time–frequency cluster (0.05–0.1 s; 8–12 Hz) in which a significant auditory gating difference was found between groups. **(C,D)** Depict the topography of S1 and S2 for the time–frequency window marked in **(A,B)**. Black boxes mark the mediocentral EEG electrode cluster, in which a significant auditory gating difference was found between groups depicted in **(A,B)**. **(E)** Illustrates the time-course of mediocentral 8–12 Hz frequency window for HC (blue) and ScZ (red). **(F)** Illustrates the significant negative correlation between auditory gating in alpha-band ITC and the PANSS negative factor for ScZ.

Sensory stimulation leads to a phase alignment shortly after stimulus onset (Cheron et al., 2007; Busch et al., 2009). Identical to the analysis of oscillatory power, the S2–S1 ITC was compared between ScZ and HC. In ScZ and HC, S2 (ScZ: 0.17 ± 0.04, HC: 0.19 ± 0.06) induced a weaker ITC than S1 (ScZ: 0.22 ± 0.05, HC: 0.33 ± 0.10), leading to a negative ITC gating difference. The auditory gating difference was stronger in HC than in ScZ, albeit in the alpha-band [8–12 Hz, *t*(42) = 3.24, *p* < 0.01, **Figures 2A–E**] in a mediocentral electrode cluster. Notably, this cluster was similar to the cluster obtained in the analysis of oscillatory power. Therefore, we chose the overlap between the clusters obtained in the analysis of oscillatory power and ITC to visualize the effects within one common region. Additional analyses revealed that alpha-band ITC was significantly stronger for HC than ScZ following S1 but not S2 (see Supplementary Material). The S2–S1 alpha-band ITC difference was distributed normally (*W* = 0.93, *p* = 0.14). In ScZ the individual alpha-band ITC gating difference was negatively correlated with the PANSS negative factor [*r*(19) = –0.54, *p* < 0.01, **Figure 2F**]. Power modulations might have contributed to this relationship (see Supplementary Material), but importantly, the correlation remained significant when controlling for medication [*r*(18) = –0.55, *p* < 0.05]. **Tables 2** and **3** summarize the main findings.

### Symptom Clusters

**FIGURE 3:**
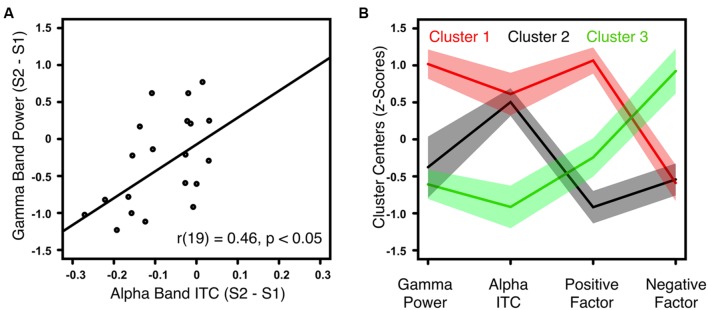
**Correlation between gamma-band power and alpha-band ITC for ScZ and outcome of the k-means cluster analysis. (A)** Illustrates the positive correlation between auditory gating (i.e., S2–S1) differences of induced gamma-band power and alpha-band ITC for ScZ. **(B)** Summarizes the symptom patterns of the three patient clusters identified by k-means clustering analysis. Positive values indicate aberrant sensory gating and high symptom scores. Negative values indicate intact sensory gating and low symptom scores. Solid lines mark the cluster centers, shaded areas mark the SEM.

The gamma-band power gating difference was positively correlated with the alpha-band ITC gating difference in HC [*r*(18) = 0.62, *p* < 0.01] and ScZ [*r*(19) = 0.46, *p* < 0.05, **Figure 3A**]. Within ScZ, the correlation remained significant when controlling for medication [*r*(18) = 0.51, *p* < 0.05]. Using the two electrophysiological markers (i.e., gamma-band power gating difference and alpha-band ITC gating difference) and the two PANSS factors (i.e., positive and negative), which were identified in the analyses above, a k-means cluster analysis was performed to identify schizophrenia phenotypes. The four-dimensional space (gamma-band power gating difference, alpha-band ITC gating difference, PANSS positive factor scores, PANSS negative factor scores) was sorted into an increasing number of clusters. The AIC identified the three-cluster solution as optimal (**Figure 3B**). Note that the values of the four markers were transformed into *z*-scores. Hence, positive values indicate aberrant sensory gating and high symptom scores. Patients in the first cluster (*N* = 7) exhibited no sensory gating (i.e., gamma-band power and alpha-band ITC to the S1 and S2 was similar), and strong positive, but low negative symptoms (**Figure 3B**, red trace). Patients in the second cluster (*N* = 6) were characterized by intact sensory gating in gamma-band power, but aberrant sensory gating in alpha-band ITC, and relatively low symptom scores (**Figure 3B**, black trace). Finally, patients in the third cluster (*N* = 8) expressed intact sensory gating in gamma-band power and alpha-band ITC, low positive symptoms but severe negative symptoms (**Figure 3B**, green trace).

## Discussion

Our study revealed that auditory sensory gating deficits relate to the schizophrenia psychopathology. While previous studies have also reported reduced sensory gating ratios in the P50, gamma-band power and alpha-band ITC (Clementz et al., 1997a; Brockhaus-Dumke et al., 2008; Patterson et al., 2008; Hall et al., 2011), a key novel finding of our study is that aberrant auditory sensory gating in gamma-band power correlates with positive symptoms in schizophrenia. Moreover, our study shows that aberrant temporal synchronization, as reflected in alpha-band phase consistency, correlates with negative symptoms. Using a cluster analysis approach, we identified three patient subgroups with distinct phenotypes of symptoms, and distinct alterations in gamma-band power and alpha-band ITC gating differences. In contrast to previous studies, we did not find differences between ScZ and HC in the auditory evoked P50 (see Supplementary Material).

### Positive Symptoms Correlate with Aberrant Gamma-Band Power Sensory Gating

In line with the literature we found that schizophrenia patients exhibit an auditory sensory gating deficit in gamma-band power (Clementz et al., 1997a; Hall et al., 2011; Popov et al., 2011, 2012). Extending previous findings, we observed that ScZ with the highest sensory gating deficits had the most severe positive symptoms. In line with our results, reduced gamma-band amplitude and impaired gamma-band sensory gating has been associated with higher perceptual anomaly (Johannesen et al., 2008). In a similar vein, previous studies reported positive relationships between gamma-band power deficits and positive symptoms in other experimental paradigms, such as the auditory oddball paradigm (Lee et al., 2003b) or visual feature binding (Lee et al., 2003a; Spencer et al., 2004). Already half a century ago, it has been proposed that sensory gating deficits could lead to an information overload that contributes to the positive symptoms in schizophrenia (McGhie and Chapman, 1961; Shakow, 1963; Freedman et al., 1991; Bob et al., 2014). In agreement with this proposal, a recent study found that auditory sensory gating deficits relate to the severity of hallucinations in schizophrenia (Smith et al., 2013). Recently, Uhlhaas and Singer (2015) proposed that positive symptoms are secondary phenomena resulting from the attempt to cope with aberrant neural synchronization, as reflected in reduced gamma-band power. Thus, aberrant neural synchronization could lead to an inability to ignore irrelevant information, which in turn could contribute to positive symptoms. Coordinated oscillatory activity in neural populations is an important process underlying cognition (Engel et al., 2001; Senkowski et al., 2008; Wang, 2010; Pittman-Polletta et al., 2015). It has been shown that GABAergic inhibition to Parvalbumin-positive (PV+) interneurons is a key element in establishing gamma-band synchrony, as it controls when neural firing takes place (Royer et al., 2012). In schizophrenia, compromised functionality of PV+ interneurons might lead to reduced gamma-band power and dysfunctional exaggerated locking of gamma-band oscillations to the trough of alpha-band oscillations (Popov and Popova, 2015).

Taken together, our study supports our hypothesis that gamma-band power gating deficits relate to positive symptoms in schizophrenia. The reduced gating in the induced gamma-band power presumably reflects the inability to inhibit irrelevant sensory information.

### Negative Symptoms are Negatively Correlated with Aberrant Alpha-Band Inter-Trial Coherence Sensory Gating

Negative symptoms are often found already in the prodromal phase of schizophrenia (Uhlhaas and Singer, 2015). These symptoms have been related to disorganized temporal synchronization (Andreasen et al., 1999). In line with this account, and in agreement with other reports (Leicht et al., 2010; Koh et al., 2011; Martin et al., 2013; Popov and Popova, 2015), we found diminished temporal synchronization as reflected in reduced phase coherence across trials in ScZ. Similar to the findings of altered gamma-band power, patients exhibited deficits in alpha-band ITC sensory gating. As previous studies have reported a reduction of alpha-band ITC (Jansen et al., 2004; Brockhaus-Dumke et al., 2008), it is somewhat surprising that schizophrenia patients who showed stronger sensory gating reported more severe negative symptoms. Previous studies have reported that negative symptoms, low levels of functioning, and reduced processing speeds are related to reduced alpha- and beta-band power (Hong et al., 2012; Smucny et al., 2013). The negative correlation between sensory gating and negative symptoms in the present study might indicate that these symptoms are related to cognitive dysfunctions and not primarily related to altered sensory processing. Our finding also seems to be in contrast with two previous studies, which reported that aberrant P50 sensory gating relates to more severe negative symptoms (Ringel et al., 2004; Thoma et al., 2005). However, these previous studies investigated the broadband evoked signal. In the present study, we analyzed the frequency-resolved signal. Hence, our finding of an alpha-band ITC gating deficit is in line with our hypothesis of reduced temporal synchronization in schizophrenia. In the prodromal phase, negative symptoms are often already present, and it is possible that altered alpha-band activity reflects a functional decline before the actual onset of the psychosis (Koh et al., 2011; Fusar-Poli et al., 2013). Interestingly, alpha-band ITC was significantly decreased in ScZ following S1. This suggests that schizophrenia patients not only suffer from an inability to inhibit irrelevant information, but also exhibit a stimulus-encoding deficit (Mathiak et al., 2011; Popov et al., 2011).

### Cluster Analysis Reveals Schizophrenia Phenotype Subgroups

An important goal in psychiatric research is to identify distinct phenotypes within the population of patients (Andreasen, 2000; Smucny et al., 2013; Boutros et al., 2014). Relating different measures of sensory gating to clinical parameters, as done in our study, can provide novel insight into the aberrant cortical processes that contribute to the schizophrenia psychopathology. To uncover possible phenotypes of schizophrenia, clinical, and cortical parameters have to be integrated. Using k-means clustering, we identified ScZ clusters with distinct deficits in oscillatory processes, and clinical symptoms. In line with the notion that positive symptoms relate to aberrant sensory gating (McGhie and Chapman, 1961; Shakow, 1963), we found one patient cluster that was characterized by a sensory gating deficit in gamma-band power and alpha-band ITC. These patients also showed pronounced positive symptoms. A second patient cluster was characterized by low positive and negative symptom scores, and intact sensory gating in the gamma-band, but aberrant gating in alpha-band ITC. Patients with aberrant temporal synchronization might suffer from cognitive deficits not limited to positive or negative symptoms (Andreasen et al., 1999; Popov and Popova, 2015). Finally, patients in the third cluster showed strong negative symptoms but relatively intact sensory gating in the alpha- and gamma-band. Negative symptoms in these patients might thus be less related to sensory gating but rather to other cognitive deficits, such as poor attention or disorientation. It is important to note that due to the low number of participants, the cluster analysis approach has an explorative character. Thus, the outcome of our analysis should be verified in a larger sample. It is also possible that group differences and correlations may be influenced by the effect of pharmacological agents on sensory gating. However, all correlations remained significant after controlling for medication. Taken together, the results of the cluster analysis underline the relationship between abnormal sensory gating in gamma-band power and positive symptoms. Conversely, abnormal gating in alpha-band ITC might relate more to unspecific symptoms in ScZ (Koh et al., 2011; Fusar-Poli et al., 2013). Our study provides novel insight into the multifaceted relationships between aberrant neural oscillations and schizophrenia psychopathology. Cluster based approaches, which integrate clinical symptoms and neural activity, seem to be suitable to characterize these relationships on an individual level.

## Summary

We related oscillatory power and temporal synchronization of time–frequency resolved EEG data to positive and negative symptoms in schizophrenia. Our study shows that the sensory gating deficit in oscillatory power is positively correlated with the positive symptoms in schizophrenia. Moreover, the sensory gating deficit in temporal synchronization is negatively correlated with negative symptoms. In addition, our cluster analysis uncovered distinct schizophrenia phenotypes. We identified three patient clusters defined by common patterns of sensory gating deficits in gamma- and alpha-band oscillations, as well as similarities in positive and negative symptoms. This shows that examining oscillatory activity enables to uncover distinct schizophrenia phenotypes. Conclusively, our study suggests that analyzing different aspects of oscillatory activity in larger cohorts, and defining specific phenotypes will substantially further our understanding of the schizophrenia psychopathology.

## Author Contributions

JK, YRR, JB, and DS designed the experiment. YRR, JB, and MH recorded the data. JK, YRR, and MH analyzed the data. JK, YRR, JB, MH, and DS wrote the manuscript.

## Conflict of Interest Statement

The authors declare that the research was conducted in the absence of any commercial or financial relationships that could be construed as a potential conflict of interest.
