# Corrigendum: Positive and Negative Symptoms in Schizophrenia Relate to Distinct Oscillatory Signatures of Sensory Gating

**DOI:** 10.3389/fnhum.2016.00162

**Published:** 2016-04-14

**Authors:** Julian Keil, Yadira Roa Romero, Johanna Balz, Melissa S. Henjes, Daniel Senkowski

**Affiliations:** Multisensory Integration Group, Department of Psychiatry and Psychotherapy, Charité - Universitätsmedizin Berlin, St. Hedwig HospitalBerlin, Germany

**Keywords:** inter-trial coherence, power, gamma-band, alpha-band, phenotype, cluster analysis

Throughout the manuscript and in the references, we have ascribed the article “Sensory disturbances, inhibitory deficits, and the P50 wave in schizophrenia” by Premysl Vlcek and colleagues erroneously to the second author, Petr Bob. Thus, references to “Bob et al., 2014” should read “Vlcek et al., 2014” and the respective reference should read:

Vlcek, P., Bob, P., and Raboch, J. (2014). Sensory disturbances, inhibitory deficits, and the P50 wave in schizophrenia. *Neuropsychiatr. Dis. Treat.* 10, 1309–1315. doi: 10.2147/NDT.S64219

## Author contributions

All authors listed, have made substantial, direct and intellectual contribution to the work, and approved it for publication.

### Conflict of interest statement

The authors declare that the research was conducted in the absence of any commercial or financial relationships that could be construed as a potential conflict of interest.

