# Supplementary material for: Positive and Negative Symptoms in Schizophrenia Relate to Distinct Oscillatory Signatures of Sensory Gating
Source: Front Hum Neurosci. 2016 Mar 14;10:104. doi: 10.3389/fnhum.2016.00104 (PMC4789458; doi:10.3389/fnhum.2016.00104)
Supplement: Supplementary file 1 [file Data_Sheet_1.DOC]

Supplementary Material to “Positive and Negative Symptoms in Schizophrenia Relate to Distinct Oscillatory Signatures of Sensory Gating.”

1. Event-related activity

1.1 Background

The auditory evoked P50, which typically peaks around 50 ms after stimulus onset, is an event-related potential (ERP) component with a fronto-central topography. The P50 originates primarily in the auditory cortex and predominantly reflects early stages of auditory processing (Clementz et al., 1997). In psychiatric research the P50 has been studied in sensory gating paradigms for more than three decades (Adler et al., 1982; see Bramon et al., 2004 for a review). Whereas the stimulation protocol of sensory gating paradigms has changed little over time, there is only little consensus regarding the data analysis (Bramon et al., 2004; Patterson et al., 2008). Moreover, the clinical correlates of sensory gating to repeated auditory stimuli are still under debate (e.g., Potter et al., 2005; Keshavan et al., 2008). One study found a correlation between thought disorder and P50 peak latency (Baker et al., 1987). Another study found a correlation between negative symptoms and P50 gating ratio (Ringel et al., 2004). In addition, a third study reported negative correlations between neuropsychological performance deficits and sensory gating (Thoma et al., 2005). Therefore, in addition to analyzing frequency-resolved oscillatory activity, we also analyzed the evoked P50 component in ScZ and HC.

1.2 Methods

1.2.1 ERP data analysis

For the analysis of the evoked P50 component, data were filtered offline with a 2 Hz, 4^th^-order, zero-phase Butterworth filter and a 45 Hz, 14^th^-order, zero-phase Butterworth filter. Averaged data was normalized relative to baseline (-0.5 s to -0.1 s baseline window). To define the P50, we selected seven fronto-central electrodes encompassing a symmetric scalp region around FCz (figure S1). In a first step, the auditory N1 component was defined as the most negative value in an interval of 0.08 s to 0.12 s after stimulus onset. Subsequently, the P50 was defined as the most positive value prior to the N1 (in accordance with (Bramon et al., 2004)). The P50 sensory gating ratio was computed using the formula: 100 x (1 – (S2 responses/S1 responses)). The P50 sensory gating difference was computed by subtracting the S1 responses from the S2 responses.

1.2.2 Statistical analysis

The P50 sensory gating ratio, as well as the P50 sensory gating difference at fronto-central electrodes were compared between ScZ and HC by means of independent-samples t-tests.

1.3 Results and Discussion

In both groups, the S2 (mean ± SD, ScZ: 0.78 ± 0.77, HC: 0.97 ± 0.83) evoked smaller P50 amplitudes than the S1 (ScZ: 0.98 ± 0.89, HC: 1.11 ± 0.78). The P50 sensory gating ratio and the P50 sensory gating difference did not differ significantly between ScZ and HC (P50 sensory gating ratio: t(42) = -0.5141, p = 0.61, P50 sensory gating difference: t(42) = -0.16, p = 0.87; see figure S1 for details). It may be that the absence of significant differences is due to the large variance of the difference values between subjects. Moreover, it is possible that the absence of effects in the evoked P50 is due to the filter settings used in our experiment. In the literature, there is little consensus on how to analyze the evoked P50 (Bramon et al., 2004; Patterson et al., 2008) and various different filter settings have been used. It is especially worrisome that some studies have applied a 10 Hz highpass filter, which could severely affect the early evoked activity (Boutros et al., 1999; Adler et al., 2004).


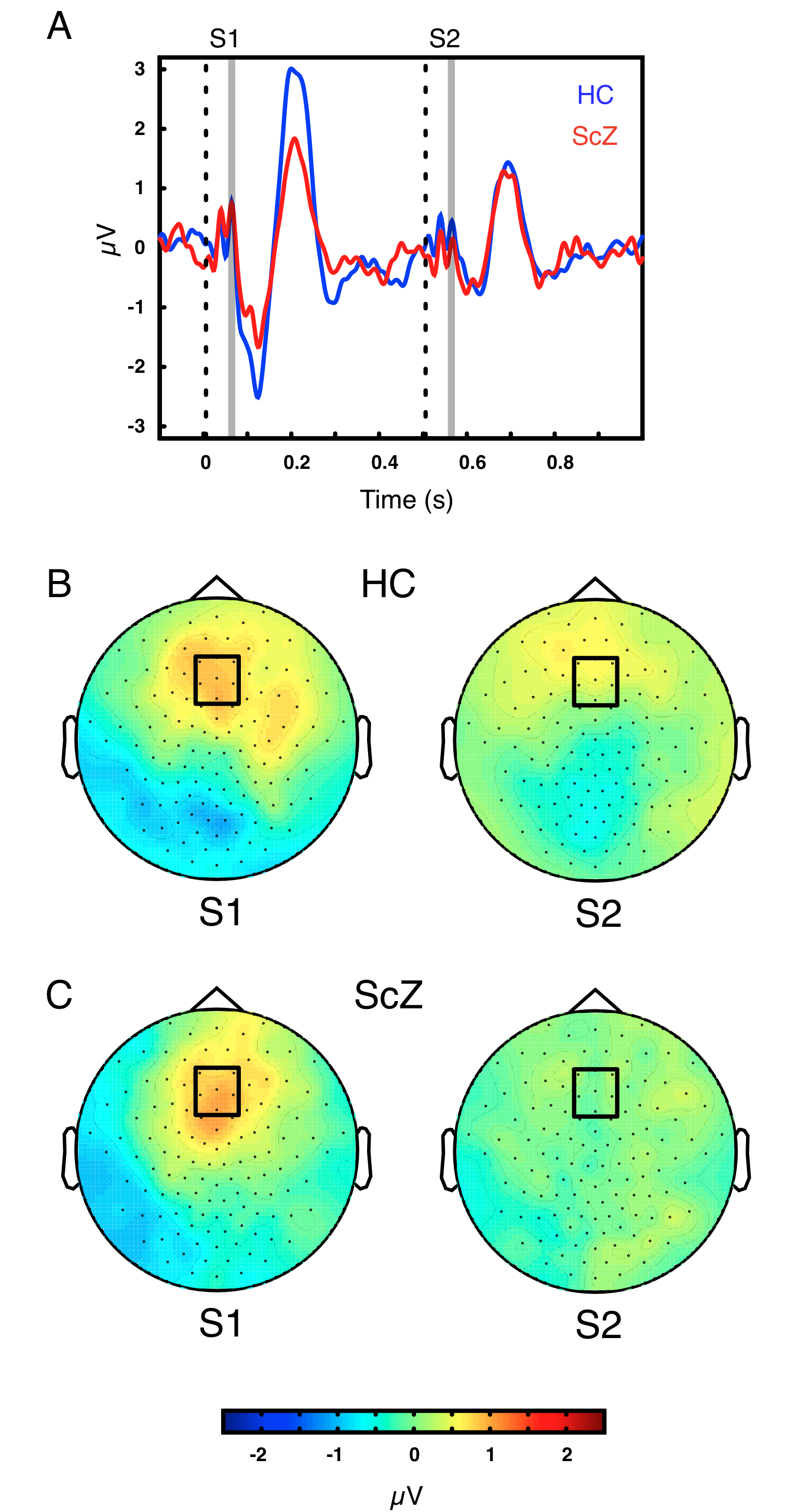


Figure S1: Activity evoked by the paired S1-S2 tones for HC and ScZ. Panel A depicts the time-course of cortical activity evoked by S1 and S2 at fronto-central electrodes for HC (blue) and ScZ (red). Dashed lines indicate the onset of auditory stimuli. Shaded areas mark the P50 components. Panel B depicts the average P50 topography for S1 (left) and S2 (right) for HC as marked by the shaded areas in panel A. Panel C depicts the average P50 topography for S1 (left) and S2 (right) for ScZ as marked by the shaded areas in panel A. Black boxes in panels B and C mark the fronto-central EEG electrodes depicted in panel A.

2. Oscillatory activity

2.1 Background

When analyzing the sensory gating between groups in terms of a S2-S1 difference, it remains unclear whether the groups also differ with respect to the actual responses to the S1 and S2. Thus, to explore whether ScZ and HC show different oscillatory activity following S1 and S2, we computed repeated-measures 2x2 factorial ANOVAs with the between-subjects factor Group (ScZ vs. HC) and the within-subjects factor Stimulus (S1 vs. S2).

2.2 Methods

2.2.1 Statistical analysis

The 2x2 factorial ANOVAs conducted for gamma-band power and alpha-band ITC were followed-up with dependent-samples t-tests in case of within-group comparisons and independent-samples t-tests in case of between-group comparisons. As four follow-up t-tests were performed, the alpha-level was Bonferroni-corrected to 0.05/4 = 0.0125.

To further elucidate the possible influence of alpha-band power modulations on the correlation between alpha-band ITC and PANSS negative symptoms (as described in the main text), the correlation between alpha-band power and PANSS negative symptoms was computed and both correlations were compared. To this end, Pearson r-values were Fisher-z transformed and compared using the formula: z = (Z_ITC_ – Z_Power_)/std(Z_ITC_ – Z_Power_) with std(Z_ITC_ – Z_Power_) = sqrt((1/n_ITC_– 3) + ((1/n_Power_ – 3)). Moreover, a partial correlation between alpha-band ITC and PANSS negative symptoms with alpha-band power as covariate was computed.

2.3 Results and Discussion

Sensory stimulation led to an increase in oscillatory power following the onset of both auditory stimuli. As described in the main text, the S2 induced weaker 30 –50 Hz gamma-band power responses than the S1 (figure S2, panel A). The repeated-measures ANOVA revealed a main effect of Group (F(1,42) = 5.86, p < 0.05), a main effect of Stimulus (F(1,42) = 20.85, p < 0.001), but only a trend towards an interaction between Group and Stimulus (F(1,42) = 3,76, p = 0.06). The follow-up t-tests revealed that stimulus induced gamma-band power tended to be stronger in HC than in ScZ following S1 (t(42) = 2.37, p = 0.02, n.s. after Bonferroni correction) and S2 (t(42) = 1.7969, p = 0.08, n.s.). Within HC stimulus induced gamma-band power was stronger to S1 compared to S2 (t(21) = 3.89, p < 0.001). Within ScZ, stimulus induced gamma-band power did not significantly differ between S1 and S2 (t(21) = 2.39, p = 0.02, n.s. after Bonferroni correction).

In addition to oscillatory power, sensory stimulation also led to an increase in alpha-band inter-trial coherence (ITC) following the onset of auditory stimulation. The repeated-measures ANOVA for alpha-band ITC revealed a main effect of Group (F(1,42) = 11.96, p < 0.005), a main effect of Stimulus (F(1,42) = 67.41, p < 0.001), and an interaction between these factors (F(1,42) = 15.39, p = 0.005). Follow-up t-tests revealed that ITC was stronger in HC than in ScZ following the S1 (t(42) = 4.25, p = 0.001) but not following the S2 (t(42) = 1.08, p = 0.29). Within both groups, alpha-band ITC was stronger in response to the S1 compared to the S2 (ScZ: t(21) = 5.28, p < 0.001, HC: t(21) = 6.64, p < 0.001). Notably, within ScZ, the alpha-band ITC in response to the S1 tended to be correlated with the PANSS negative factor (r(19) = 0.38, p = 0.09).


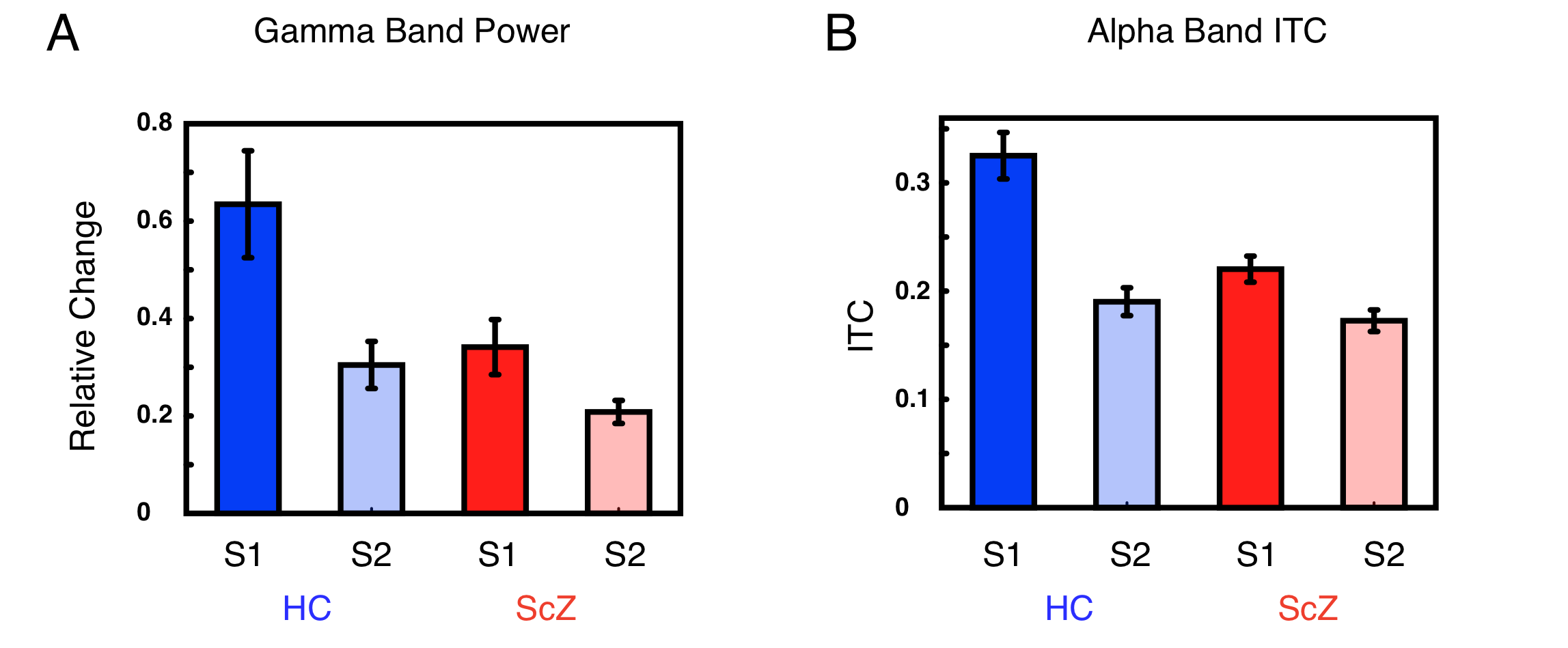
Figure S2: Average oscillatory activity following S1 (dark colors) and S2 (light colors) for HC (blue) and ScZ (red). Panel A depicts the average 30 – 50 Hz gamma-band power in the mediocentral electrode cluster. Panel B depicts the average 8 – 12 Hz alpha-band ITC.

As reported in the main text, the alpha-band ITC S2-S1 gating difference was negatively correlated with the PANSS negative factor. Interestingly, the alpha-band power S2-S1 gating difference also correlated negatively with the PANSS negative factor (r(19) = - 0.45, p = 0.04). However, since PANSS symptom scores were grouped into five factors, this correlation does not survive the Bonferroni-corrected alpha-level of 0.05/5 = 0.01. To further examine the influence of stimulus induced power modulations on the correlation between alpha-band ITC and the PANSS negative factor, both correlations were compared, but the correlations did not significantly differ (z = 0.3579). In addition, the partial correlation analysis between the S2-S1 sensory gating difference in alpha-band ITC and PANSS negative factor with alpha-band power as covariate was not significant (r(18) = -0.33, p = 0.15). Taken together, this suggests that alpha-band power modulations contributed, at least in part, to the observed relationship between alpha-band ITC and the schizophrenia psychopathology.

References:

Adler, L. E., Olincy, A., and Cawthra, E. M. (2004). Varied effects of atypical neuroleptics on P50 auditory gating in schizophrenia patients. American Journal of Psychiatry 161, 1822-1828

Adler, L. E., Pachtman, E., Franks, R. D., Pecevich, M., Waldo, M. C., and Freedman, R. (1982). Neurophysiological evidence for a defect in neuronal mechanisms involved in sensory gating in schizophrenia. Biol. Psychiatry 17, 639–654.

Baker, N., Adler, L. E., Franks, R. D., Waldo, M., Berry, S., Nagamoto, H., et al. (1987). Neurophysiological assessment of sensory gating in psychiatric inpatients: comparison between schizophrenia and other diagnoses. Biol. Psychiatry 22, 603–617.

Boutros, N. N., Belger, A., Campbell, D., D'Souza, C., and Krystal, J. (1999). Comparison of four components of sensory gating in schizophrenia and normal subjects: a preliminary report. Psychiatry Research 88, 119–130.

Bramon, E., Rabe-Hesketh, S., Sham, P., Murray, R. M., and Frangou, S. (2004). Meta-analysis of the P300 and P50 waveforms in schizophrenia. Schizophrenia Research 70, 315–329. doi:10.1016/j.schres.2004.01.004.

Clementz, B. A., Geyer, M. A., and Braff, D. L. (1997). P50 suppression among schizophrenia and normal comparison subjects: a methodological analysis. Biol. Psychiatry 41, 1035–1044. doi:10.1016/S0006-3223(96)00208-9.

Keshavan, M. S., Tandon, R., Boutros, N. N., and Nasrallah, H. A. (2008). Schizophrenia, “just the facts”: what we know in 2008 Part 3: neurobiology. Schizophrenia Research 106, 89–107. doi:10.1016/j.schres.2008.07.020.

Patterson, J. V., Hetrick, W. P., Boutros, N. N., Jin, Y., Sandman, C., Stern, H., et al. (2008). P50 sensory gating ratios in schizophrenics and controls: A review and data analysis. Psychiatry Research 158, 226–247. doi:10.1016/j.psychres.2007.02.009.

Potter, D., Summerfelt, A., Gold, J., and Buchanan, R. W. (2005). Review of Clinical Correlates of P50 Sensory Gating Abnormalities in Patients with Schizophrenia. Schizophr Bull 32, 692–700. doi:10.1093/schbul/sbj050.

Ringel, T. M., Heidrich, A., Jacob, C. P., and Fallgatter, A. J. (2004). Sensory gating deficit in a subtype of chronic schizophrenic patients. Psychiatry Research 125, 237–245. doi:10.1016/j.psychres.2004.01.004.

Thoma, R. J., Hanlon, F. M., Moses, S. N., Ricker, D., Huang, M., Edgar, C., et al. (2005). M50 sensory gating predicts negative symptoms in schizophrenia. Schizophrenia Research 73, 311–318. doi:10.1016/j.schres.2004.07.001.
